# ESPO AND BUTLER-WILLIAMS SCIENTIFIC SYMPOSIUM: CAREER DEVELOPMENT TO PROMOTE DIVERSITY, DISCOVERY, AND AGING

**DOI:** 10.1093/geroni/igac059.215

**Published:** 2022-12-20

**Authors:** Kalisha Bonds Johnson, Brianna Morgan, Patricia Jones

## Abstract

The NIA’s Butler-Williams Scholars Program and GSA’s ESPO Section are united in providing career development opportunities in a manner that promotes leadership, diversity, and inclusivity. This year’s theme challenges our emerging scholars to embrace diversity and discovery while thinking—or rethinking—about the perspectives of older adults. Disparities in health associated with race/ethnicity, experience, sociocultural and socioeconomic factors, as well as access to and provision of health care are chief concerns of our aging population. GSA’s early career professionals and 2021 alumni of the prestigious NIA Butler-Williams Scholars Program address these issues. Dr. Matthew Farina will present on the importance of identifying life course pathways in understanding the lived experiences of older adults from underrepresented racial groups. Dr. Kacie Deters will discuss how both demographics and genetic factors contribute to cognitive performance. Dr. Mirna Arroyo-Miranda will present findings on social isolation and cognition in Hispanic/Latino older adults. The final speaker, Dr. Jamaine Davis will present on the genetic factors that may contribute to dementia in African American older adults. The featured talks by rising stars deepen our understanding of the influence of diversity and key discoveries so we can reimagine aging research.

